# Plasma Brain-Derived Neurotrophic Factor (BDNF) Levels and BDNF Promoters’ DNA Methylation in Workers Exposed to Occupational Stress and Suffering from Psychiatric Disorders

**DOI:** 10.3390/brainsci14111106

**Published:** 2024-10-30

**Authors:** Antonello Veltri, Vanessa Nicolì, Riccardo Marino, Filomena Rea, Martina Corsi, Martina Chiumiento, Marianna Giangreco, Fabrizio Caldi, Giovanni Guglielmi, Rudy Foddis, Fabio Coppedè, Roberto Silvestri, Rodolfo Buselli

**Affiliations:** 1 Center for Work-Related Stress and Occupational Mental Disorders, Azienda Ospedaliero-Universitaria Pisana, 56124 Pisa, Italy; 2 Occupational Health Unit, Azienda Ospedaliero-Universitaria Pisana, 56124 Pisa, Italy; 3 Department of Translational Research and of New Surgical and Medical Technologies, University of Pisa, 56126 Pisa, Italy; 4 Department of Surgical, Medical, Molecular and Critical Area Pathology, University of Pisa, 56124 Pisa, Italy; 5 Department of Biology, University of Pisa, 56126 Pisa, Italy

**Keywords:** brain-derived neurotrophic factor, BDNF exon I and IV promoters’ methylation, epigenetic changes, work-related stress, adjustment disorders, major depressive disorder

## Abstract

Introduction: Decreased plasma BDNF (pBDNF) levels have been proposed as a biomarker in the illness phases of mood disorders. This cross-sectional study aimed to evaluate the pBDNF and BDNF promoters’ DNA methylation levels in workers exposed to occupational stress and suffering from work-related stress disorders. Methods: the pBDNF and BDNF exon I and IV promoters’ methylation levels were measured by specific immunoassays and methylation-sensitive high-resolution melting (MS-HRM) in 62 patients with adjustment disorders (AD), 79 patients with major depressive disorder (MDD) and 44 healthy controls. Occupational stress was evaluated in the patients and controls using the Job Content Questionnaire (JCQ). Results: the pBDNF levels were significantly higher in the MDD (*p* < 0.001) and AD (*p* < 0.0001) patients than in the controls. The MDD patients showed significantly lower pBDNF levels than the AD ones (*p* = 0.01). The BDNF exon I and IV promoters’ methylation levels were significantly higher in the MDD patients than in the AD ones (exon I promoter: *p* = 0.0001, exon IV promoter: *p* < 0.0001) and controls (exon I promoter: *p* = 0.0001, exon IV promoter: *p* < 0.0001). In the patients, but not in the controls, the BDNF promoters’ methylation levels showed significant negative correlations with occupational stress. Conclusions: BDNF could play a key role in the pathophysiology of stress-related disorders and the peripheral elevation of it observed in patients exposed to occupational stress could suggest a protective mechanism for neurons from stress-mediated damage. The elevation of the pBDNF levels, even in MDD, may characterize a “reactive” subtype of depressive episode, while the significant elevation of the BDNF promoters’ methylation levels in depressed patients could indicate a predisposition to more severe illness under stress. Further research is needed, focusing on biomarkers for stress-related disorders as a potential tool for the diagnosis and prevention of occupational diseases.

## 1. Introduction

Work-related stress is an increasing risk in occupational medicine, with a deep impact on workers’ performance and global health [1,2]. Exposure to chronic work-related stress could lead to psychiatric occupational diseases such as adjustment disorders (AD), and this increasingly determines the need for the integration of tools and skills from occupational medicine and psychiatry [3,4]. AD occur within three months after one or more objectively identified stressful events and do not persist for more than an additional 6 months once the stressor, or its consequences, terminates. They are characterized by heterogeneous emotional or behavioral symptoms belonging to the anxiety area or to the depressive one, causing significant impairment in social, occupational, or other important areas of functioning [5]. Despite being crucial for public insurance issues, the differential diagnosis between major depressive disorder (MDD), above all if mild/moderate, and AD is often not simple [6,7].

The neurotrophic hypothesis posits that stress and depression are correlated with a deficit in neurotrophins, which leads to neuronal atrophy and cell loss in crucial brain regions, including the limbic system and the prefrontal cortex [8]. Among the neurotrophins, brain-derived neurotrophic factor (BDNF) has drawn particular attention due to its role as an allostatic mediator, essential for supporting memory, learning, and the brain’s adaptive responses to environmental changes [9]. In response to chronic stress, proinflammatory cytokines (such as TNF-α, IL-6, and IL-1β) and glucocorticoids exert a repressive effect on the BDNF gene, contributing to brain atrophy and the development of mood disorders in susceptible individuals [10]. This repressive effect on BDNF gene expression is, in part, mediated through epigenetic mechanisms such as DNA methylation. Numerous studies have shown that the peripheral (serum and/or plasma) BDNF levels are lower in patients with mood disorders during manic/mixed and depressive episodes compared to matched healthy controls, and that effective treatments can normalize these levels [11,12,13,14,15,16,17,18,19,20]. These findings suggest that the peripheral BDNF levels could serve as potential biomarkers of mood (depressive and manic/mixed) episodes, as well as predictors of treatment efficacy.

DNA methylation, particularly in the promoter regions of genes, is a well-established mechanism for silencing gene expression. With regard to BDNF, the exon IV promoter region has been extensively studied in both animal and human models. Research in rodents has demonstrated that exposure to early-life adversity or chronic stress leads to increased methylation of the BDNF exon IV promoter, which is associated with decreased BDNF expression in the prefrontal cortex—a region heavily implicated in emotional regulation and cognitive function [21]. This epigenetic modification appears to persist long after the initial stressor, suggesting that BDNF gene silencing through methylation could contribute to long-term vulnerability to psychiatric disorders. At present, a growing body of data has associated the methylation of BDNF promoters in human peripheral blood cells with the development of various neuropsychiatric disorders. Most of the studies have analyzed the BDNF exon I and IV promoters, producing contrasting results [22]. Higher methylation levels of BDNF promoters have been described in patients with major depression or exposed to psychosocial stress compared to healthy controls and found to be associated with a previous suicidal attempt history [23,24,25], with suicidal ideation during treatment [26], and with the response to antidepressant treatments [27,28].

Despite substantial evidence supporting the role of BDNF in mood disorders, there remain unresolved questions regarding the relationship between BDNF levels, its gene regulation (specifically through methylation) and psychiatric outcomes in the context of work-related stress. The inconsistencies in the studies on BDNF promoters’ methylation levels in psychiatric patients underscore the need for further investigation into the role of BDNF methylation in different psychiatric phenotypes. Given these gaps in the literature, the rationale for our study was to explore whether BDNF levels and methylation patterns could differentiate between workers exposed to occupational stress and suffering from psychiatric disorders compared to healthy controls. Our central hypothesis was that workers experiencing significant occupational stress and presenting psychiatric disorders will exhibit altered plasma BDNF (pBDNF) levels and increased methylation of BDNF promoters, reflecting stress-related epigenetic regulation. Moreover, we hypothesized that pBDNF levels and BDNF DNA methylation may correlate with the severity of psychiatric symptoms, potentially serving as biological markers of stress-related psychiatric disorders in occupational settings. Therefore, the aim of the present study was to measure pBDNF levels and BDNF exon I and IV promoters’ methylation levels in workers exposed to occupational stress and afferent to a center for work-related stress and occupational mental health. In particular, we aimed to investigate workers suffering from typical stress-related psychiatric disorders, such as AD, but also those who developed MDD during exposure to occupational stress, compared to healthy control subjects. We also investigated the possible correlations between the biological variables and patients’ clinical characteristics.

## 2. Methods

### 2.1. Subjects

The present study has a cross-sectional design and examines two clinical samples consisting of, respectively, 62 AD patients (32 M—51.6%, mean age ± SD: 47.6 ± 8.4 years) and 79 MDD patients (33 M—41.8%, mean age ± SD: 50.3 ± 8.0 years) consecutively recruited at the Center for Work-Related Stress and Occupational Mental Disorders operating in the Azienda Ospedaliero-Universitaria Pisana. A control group of 44 healthy workers (26 M—51.9%, mean age ± SD = 47.1 ± 8.5 years) was recruited from blood donors in the hospitals of Tuscany and Liguria.

The AD patient group included cases of different clinical AD subtypes according to the DSM-5 classification (with depressed mood, with anxiety, with mixed anxiety and depressed mood, with disturbance of conduct, with mixed disturbance of emotions and conduct, unspecified). The MDD group included patients suffering from major depressive disorder according to the DSM-5 criteria. The exclusion criteria for patients were as follows: age lower than 18 or higher than 65 years, presence of comorbid major neurological or medical illnesses, current acute or chronic inflammatory diseases in treatment with steroidal anti-inflammatory drugs, pregnancy, presence of other current comorbid psychiatric disorders, presence of lifetime psychiatric comorbidity, onset of current symptoms as a reaction to preceding stressors different from current stressful work situations, previous recurrent mood episodes (for the MDD group), insufficient comprehension of Italian that prevented completion of self-report questionnaires, and inability to sign informed consent. To avoid a potential effect of treatment changes on the central and peripheral BDNF levels, those AD and MDD patients who were taking psychopharmacological treatments were excluded if changes in their drug treatment had been necessary in the last 8 weeks before the blood collection.

The exclusion criteria for the healthy controls were as follows: age lower than 18 or higher than 65 years, history of past and/or current major medical or mental disorders, heavy cigarette smoking, current treatment with steroidal anti-inflammatory drugs, regular medication intake and/or substance abuse.

This research project was approved by the Ethics Committee of Area Vasta Nord Ovest of Tuscany according to the Declaration of Helsinki (2013), where the approval code is 204/2014 from 12 April 2014, and participation in the study was formalized with the collection of written informed consent.

### 2.2. Clinical Assessment

Patients diagnosed with AD were subjected to a medical examination conducted by an occupational physician, complemented by psychiatric and psychological assessments. The medical examination, coupled with the collection of the medical history and review of each patient’s clinical records, allowed the exclusion of individuals with neurological or medical conditions. Following this, the patients were asked to complete a series of questionnaires and rating scales.

A self-report form was used to compile sociodemographic and employment-related information, encompassing aspects such as the nature of the contract, duration of employment in the current and previous workplaces, and the size and sector of the organization.

The clinical diagnoses of AD and MDD were validated using the Structured Clinical Interview for DSM-5 Clinician Version (SCID-5-CV) [29]. This tool, in conjunction with the psychiatric clinical evaluation, allowed the exclusion of patients with other concurrent comorbid psychiatric disorders, history of recurrent depressive episodes, or lifetime psychiatric comorbidity.

The assessment of work-related stress was conducted using the Job Content Questionnaire (JCQ), a widely utilized self-administered questionnaire designed to gauge the social and psychological aspects of jobs [30]. It includes 49 items for which the responses are typically given on a Likert scale, ranging from “strongly disagree” to “strongly agree”. The JCQ’s most recognized scales, including decision latitude, psychological job demands, and social support, are employed to measure the high-demand/low-control/low-support model of job strain development. In particular, the decision latitude scale assesses the extent to which a worker can make decisions and exercise control over their work (decision authority and skill discretion); the psychological job demand scale measures mental arousal or stimulation associated with task completion; and the social support scale examines the degree of psychological and physical assistance provided at work by supervisors/coworkers [30]. In this study, we used the Italian version of the questionnaire [31].

The Beck Depression Inventory-II (BDI-II), a widely adopted 21-item multiple-choice self-report test with two subscales (cognitive-affective and somatic), was administered to the AD and MDD patients to evaluate the severity of their depressive symptoms [32]. The internal consistency of the Italian version of the BDI-II measured by Cronbach’s alpha is 0.86 for the first factor and 0.65 for the second factor [33].

Anxiety in the AD and MDD patients was assessed using the Self-rating Anxiety Scale (SAS) [34]. It is a 20-item self-report assessment tool built to measure state anxiety levels. It has a good internal consistency, as demonstrated by the Cronbach’s alpha of 0.86. The raw scores range from 20 to 80. The best cut-off was proposed to be 40 for clinical settings and 36 for screening purposes [35]. The instrument is suited to investigating anxiety disorders and shows strong correlations with other similar instruments [36]. In this study, we used the Italian version [37].

The healthy workers were requested to complete the self-report form for sociodemographic and work information, and the JCQ. Psychiatric diagnoses in the controls were excluded through a psychiatric evaluation. Medical comorbidity and substance abuse were also ruled out in the healthy controls through a comprehensive medical examination, collection of the medical history, and review of clinical records.

### 2.3. Measurement of pBDNF Through Enzyme-Linked Immunosorbent Assay

To avoid a potential bias due to the diurnal rhythm of the pBDNF levels [38], venous blood samples were drawn in the morning (between 8:00 and 10:00 a.m.) and collected into EDTA-coated tubes. For the plasma extraction, the samples were centrifuged at 3000× *g* for 10 min at 4 °C and the supernatant was retrieved and stored at −80 °C. EDTA was chosen as the anticoagulant to avoid heparin interference with the enzyme-linked immunosorbent assay (ELISA) [39]. The pBDNF concentration was measured using an ELISA assay according to the manufacturer’s instructions (Abcam BDNF Human ELISA kit, Cambridge, UK). Before carrying out the assay, each plasma sample was diluted 1:100 into an appropriate buffer provided with the kit.

### 2.4. Gene-Specific Methylation Analysis

Peripheral blood was sampled to extract genomic DNA using the QIAamp^®^ DNA Blood Mini Kit (QIAGEN, Milan, Italy), following the manufacturer’s protocol. Thus, the genomic DNA was quantified using a Nano Drop ND 2000c spectrophotometer (Thermo Fisher Scientific, Milan, Italy) and 200 ng was treated with sodium bisulfite using the “EpiTect^®^ Bisulfite Kit” (QIAGEN, Milan, Italy). During the bisulfite treatment, unmethylated cytosines were initially deaminated and subsequentially converted into thymine following a PCR amplification, while methylated cytosines were preserved by methyl group.

The DNA methylation analysis was performed by means of the methylation-sensitive high-resolution melting (MS-HRM) technique with a CFX96 Real-Time PCR detection system (Bio-Rad, Milan, Italy). In-house MS-HRM protocols were developed for each promoter region using specific methylation-independent primers designed with the MethPrimer software version 1.0 (Table 1). In particular, the MS-HRM analysis included an initial denaturation PCR step (95 °C, 3 min), followed by 50 cycles including denaturation (95 °C, 30 s), annealing (30 s at primer-specific temperature) and elongation (72 °C, 30 s). An additional denaturation and a renaturation step (50 °C, 1 min) were performed before the last HRM step (temperature increase from 65 °C to 95 °C (0.2 °C every 15 s).

**Figure 1:**
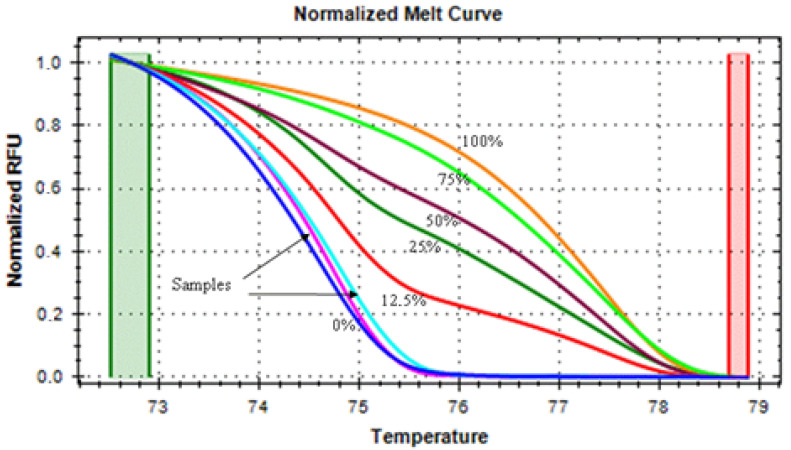
Melting curves of the PCR products of the BDNF-IV gene. The melting curves were generated by samples with known 0% (dark blue curve), 12.5% (red curve), 25% (dark green curve), 50% (dark violet curve), 75% (light green curve), and 100% (orange curve) methylation levels, obtained by mixing the fully methylated and unmethylated standard DNA samples. The curves indicated by the arrows represent the melting curves of two samples (light violet and light blue curves).

**Figure 2:**
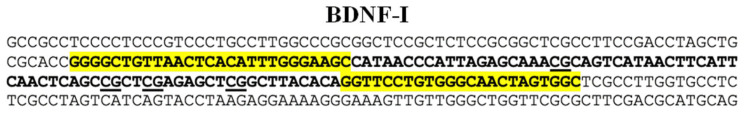
BDNF-I region analyzed/DNA sequence from NCBI Reference Sequence: NG_011794.1, nucleotides 4557 to 4675. In yellow, binding sequences of the primers used in the present study for the MS-HRM experiments. In bold, the amplicon analyzed in the present study with the underlined CpG sites.

**Figure 3:**
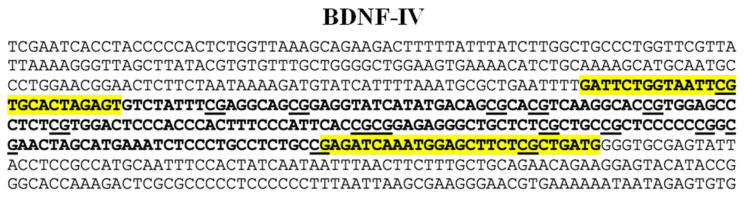
BDNF-IV region analyzed/DNA sequence from NCBI Reference Sequence: NG_011794.1, nucleotides 25326 to 25537. In yellow, binding sequences of the primers used in the present study for the MS-HRM experiments. In bold, the amplicon analyzed in the present study with the underlined CpG sites.

Standard samples with known methylation percentages of 0, 12,5, 25, 50, 75, and 100 (Qiagen, Milan, Italy) (Figure 1) were included in each experiment in order to extrapolate the methylation level of the tested DNA, as previously detailed in PMID: 33192293. Each experiment was performed in duplicate. Figure 2 and Figure 3 display the nucleotide sequences with the marked CpG sites for each of the examined BDNF regions (exons I and IV).

### 2.5. Statistical Analysis

The data were recorded in a specifically designed database and elaborated by means of the MedCalc software (version 12.7). According to the Kolmogorov–Smirnov test, all the examined continuous variables were non-Gaussian distributed, except age. Therefore, the comparisons between groups of variables with a non-Gaussian distribution were performed by means of non-parametric statistical tests; in particular, the Mann–Whitney test and the Kruskal–Wallis test were used. For the comparison of groups with Gaussian distributed variables, the one-way ANOVA was performed. The Chi-square test was used to compare the frequencies of categorical variables. The correlations between continuous variables with a non-Gaussian distribution were examined by means of Spearman’s coefficient. A *p* value less than 0.05 was considered significant.

## 3. Results

### 3.1. Comparisons Between Patients and Controls

The sociodemographic and work characteristics, pBDNF and BDNF exon I and IV promoters’ methylation levels, and rating scale scores of the three groups (AD patients, MDD patients and healthy controls) are reported in Table 2.

The three groups did not differ significantly in terms of their age, sex, and education level. Most AD patients (61.3%) and controls (73.2%), and about half of the MDD patients (48.1%), were working for private companies. The frequency of having a stable job was significantly higher in the patient groups (AD: 98.4%, MDD: 92.2%) than in the control one (78.0%, *p* = 0.0001).

**Figure 4:**
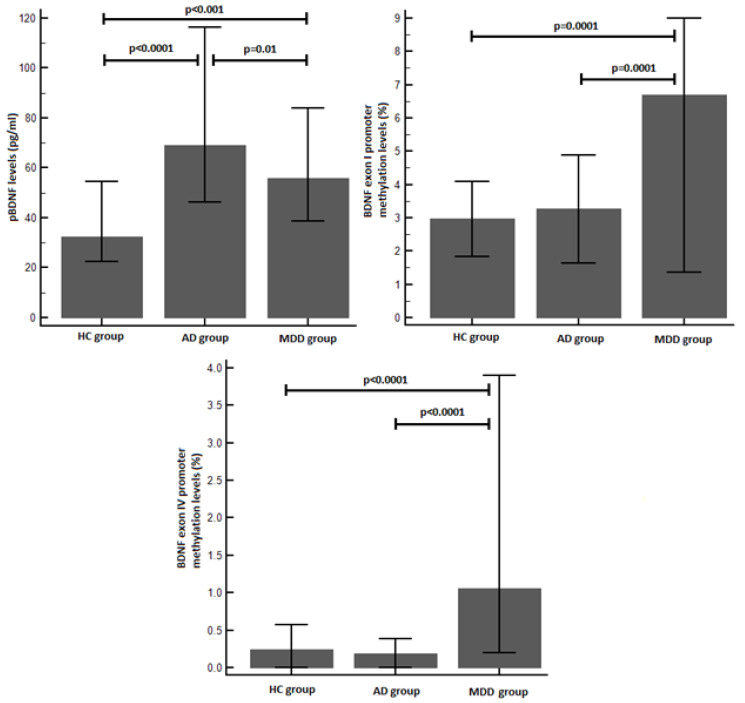
Post hoc comparisons between patients and healthy controls for the pBDNF and BDNF exon I/IV promoters’ methylation levels. Legend—AD: adjustment disorder; MDD: major depressive disorder; HC: healthy control; pBDNF: plasma brain-derived neurotrophic factor.

The pBDNF levels were significantly different between groups (*p* < 0.000001). Post hoc analyses demonstrated higher pBDNF levels both in the AD (*p* < 0.0001) and MDD groups (*p* < 0.001) compared to the control group. Moreover, the MDD patients showed lower pBDNF levels than the AD ones (*p* = 0.01) (Figure 4).

Similarly, we found significant differences between the groups in terms of DNA methylation. Post-hoc analyses showed significantly higher levels of BDNF exon I and IV promoters’ methylation in the MDD group than in the AD (exon I promoter: *p* = 0.0001, exon IV promoter: *p* < 0.0001) and control (exon I promoter: *p* = 0.0001, exon IV promoter: *p* < 0.0001) groups (Figure 4).

No significant differences were found between the MDD and the AD groups for the BDI-II and SAS scores.

The three groups significantly differed in terms of the JCQ job demands (*p* < 0.000001), social support (*p* < 0.000001) and decision latitude (*p* < 0.001) scale scores. In particular, post-hoc analyses for the JCQ job demands scale score demonstrated higher scores in both the MDD (*p* < 0.001) and AD (*p* < 0.001) groups than in the control group. The MDD patients showed higher scores than the AD ones (*p* < 0.0001). Post hoc analyses for the JCQ job social support scale score revealed lower scores in the AD group than in both the MDD (*p* < 0.0001) and control ones (*p* < 0.0001). Lastly, post hoc analyses for the JCQ decision latitude scale score highlighted higher scores in the control group than in both the MDD (*p* = 0.0001) and AD (*p* < 0.01) ones.

### 3.2. Correlation Analyses

The results of the correlation analyses between the clinical and biological variables in the three groups are reported in Table 3.

No significant correlations were found between the pBDNF levels, JCQ scales scores, BDI-II and SAS scores in all three groups.

As regards the BDNF exon I and IV promoters’ methylation levels, no correlations were found with the BDI-II and SAS scores in all three groups. In the AD patients, a significant negative correlation was only found between the BDNF exon IV promoter’s methylation levels and JCQ job demands scale score (r = −0.295, *p* = 0.02). In the MDD group, the BDNF exon I promoter’s methylation levels were negatively correlated with both the JCQ job demands scale score (r = −0.236, *p* = 0.04) and JCQ social support scale score (r = −0.244, *p* = 0.03). Moreover, in the MDD group, the BDNF exon IV promoter’s methylation levels also negatively correlated with both the JCQ job demands scale score (r = −0.588, *p* < 0.0001) and JCQ social support scale score (r = −0.686, *p* < 0.0001). In the healthy controls, a significant positive correlation was only found between the BDNF exon I promoter’s methylation levels and JCQ job demands scale score (r = 0.374, *p* = 0.01).

## 4. Discussion, Limitations and Conclusions

As expected, the group of AD and MDD patients recruited at the Center for Work-Related Stress and Occupational Mental Disorders operating in the University Hospital of Pisa showed higher levels of occupational stress than the healthy workers. In particular, based on the JCQ questionnaire, the AD patients’ jobs appeared to be characterized by greater job demands, lower social support and less decision latitude compared to those of the controls. The MDD patients’ jobs showed higher job demands and lower decision latitude than the healthy workers. Workers suffering from mental disorders were more stressed despite having more favorable working conditions (almost all the patients had a stable job) compared to the controls.

In the present study, the pBDNF levels were significantly higher in the AD and MDD patients than in the healthy workers. The data regarding the pBDNF levels in the AD patients confirm a previous observation that allows some speculative hypotheses. The higher pBDNF levels in the AD patients than the controls could be an expression of the initial compensatory neuroprotective mechanisms from stress [40]. This mechanism could be similar to that observed in other disorders, such as fibromyalgia (FM), in which both the plasma and serum BDNF levels were reported to be higher in patients than healthy controls, leading researchers to hypothesize that BDNF increases in FM patients because it is involved in the compensatory modulatory mechanisms of pain [41,42]. Some preclinical studies support the hypothesis of an initial pBDNF increase as a homeostatic mechanism following stress exposure. For instance, an increase in BDNF gene expression has been reported in various regions of the rat brain (hippocampus, amygdala, cortex) following different types of stress (maternal separation, social defeat, acute and chronic restraint) [43]. Additionally, an elevation in the pBDNF levels has been demonstrated in rats exposed to both acute and chronic stress [44,45]. Conversely, the observation of higher pBDNF levels in our patients with MDD compared to the healthy controls is more challenging to interpret. As documented extensively in the literature, the peripheral blood (plasma and/or serum) BDNF levels are typically lower in patients suffering from mood disorders during manic, mixed, or depressive episodes when compared to matched healthy controls. Furthermore, effective treatments for these disorders have been shown to normalize the BDNF levels, suggesting that BDNF could serve as a biological marker of disease activity and therapeutic response [11,12,13,14,15,17,18,19,20]. The divergence observed in our study may be explained by the “type” of depressive episode we analyzed—specifically, first episodes of MDD that are chronologically associated with psychosocial stressors. This aligns with the well-established “kindling” hypothesis, which posits that psychosocial stressors play a more significant role in triggering the first episode of major depressive disorder compared to subsequent episodes [46]. In this context, our cohort may reflect a different biological response to depression, particularly in what is referred to as “reactive depression” [47], where the episode is closely linked to identifiable life stressors. One possible explanation for the elevated pBDNF levels in this specific subtype of MDD could be an initial compensatory neurobiological response aimed at repair and resilience. This is similar to what has been observed in AD, where BDNF-mediated neuroplasticity is thought to represent an attempt to counteract the detrimental effects of stress on brain function. In this light, the increase in pBDNF may indicate an early phase of BDNF-driven neural repair, which could differentiate first depressive episodes from the more chronic forms of the disorder, where the BDNF levels are consistently lower. Further research is needed to explore whether this BDNF elevation is transient, representing an initial protective factor in the early stages of MDD. Such findings could have important implications for tailoring treatment strategies based on the stage and nature of the depressive episode.

Our analyses revealed a significant negative correlation between job demands and the methylation levels of the BDNF exon I (in MDD) and IV (in MDD and AD) promoters in patients with psychiatric disorders, particularly in those with major depressive disorder (MDD). These findings may support the “reactivity hypothesis”, which posits that work-related stress, through epigenetic mechanisms, could trigger a compensatory protective response in individuals with psychiatric conditions. Specifically, stress may “unmute” the BDNF gene by reducing methylation in critical promoter regions, leading to increased BDNF production and release as a neuroprotective response. This aligns with the notion that heightened stress exposure in vulnerable individuals may activate biological pathways aimed at promoting resilience and neural repair. To the best of our knowledge, no previous study has reported such a negative correlation between occupational stress and BDNF promoter’s methylation in psychiatric patients. In the healthy controls, on the other hand, we observed a positive correlation between job demands and the methylation levels of the exon IV promoter. This observation is in line with other previous reports. Song et al. (2014) reported increased methylation levels of the whole BDNF gene in highly stressed workers compared to those exposed to low levels of job stress [48]. Bakusic et al. (2020) found significantly increased methylation of the BDNF exon I and IV promoters in a sample of workers with burnout, which also correlated with their burnout symptoms [25]. These findings highlight the complex interplay between job stress, BDNF methylation, and psychiatric status, and they underscore the importance of considering individual differences in stress reactivity when examining the epigenetic regulation of stress-related genes. Future studies should investigate whether these epigenetic changes are reversible with intervention or whether they represent a stable, long-term adaptation to occupational stress.

Another point is that the pBDNF levels in our MDD patients, although significantly higher than those of the controls, were significantly lower than those of AD patients. The methylation levels were significantly and abnormally higher in the MDD patients compared to the AD patients and controls. This observation could suggest the existence, in MDD patients, of some mechanism (which perhaps expresses an individual predisposition to become more severely ill under stress) associated with abnormally high levels of methylation of BDNF promoters. This could gradually cause the depletion of BDNF and neuroplasticity impairment up to brain atrophy, which are largely reported in mood disorders but also in post-traumatic stress disorder (PTSD) and burnout cases [49,50,51]. It can be hypothesized that, over time, the BDNF levels in our group of MDD patients would decline further. It is clear that a longitudinal study would be needed to explore this hypothesis. In any case, the finding of higher methylation levels of BDNF promoters in depressed patients compared to healthy controls is consistent with previous research [23,24,52]. Na et al. (2016) also reported an inverse correlation between the levels of BDNF promoters’ methylation and the cortical thickness of different brain regions among patients with MDD [52].

This study is not without limitations that warrant mention. The first limitation of our study is its cross-sectional design, which restricts our ability to draw conclusions about the causality between the variables. While this design allows us to identify associations and generate hypotheses, it does not enable us to determine the directionality of these relationships or establish cause-and-effect links. We acknowledge that future longitudinal studies are needed to better understand the temporal dynamics of BDNF changes and their relationship with psychiatric disorders. Longitudinal research would also enable the examination of potential mediating or moderating factors—such as stress exposure or genetic predispositions—that could influence the relationship between BDNF and psychiatric disorders. The second potential limitation of this study is the relatively small sample size, particularly in the control group. While the sample is adequate for the scope of the research and the three groups are comparable in terms of their age, gender, and education, caution should be exercised when generalizing these findings to broader populations. Future research with larger samples is encouraged to validate and extend the applicability of our results. A third limitation of our study is the heterogeneity within the AD group in terms of the clinical subtypes. AD present with a range of emotional and behavioral symptoms that can vary significantly across individuals. This variability may have influenced our findings, as different subtypes could be associated with distinct biological mechanisms, including variations in the BDNF levels and DNA methylation patterns. Future studies on larger samples should consider stratifying patients based on the AD subtypes to better understand how these differences might impact the biological markers investigated, allowing for more accurate and clinically meaningful conclusions. Another limitation of our study is the underlying assumption that the blood BDNF levels serve as a significant reflection of the brain BDNF concentrations. While there is ongoing debate in the literature regarding the extent to which the peripheral BDNF levels mirror central nervous system (CNS) function, several lines of evidence suggest a plausible connection. BDNF is known to traverse the blood–brain barrier [53], and studies in rodent models have shown positive correlations between changes in the central and peripheral BDNF levels [54]. This supports the hypothesis that circulating BDNF may contribute to neuroprotection and the maintenance of neural cell function. However, caution must be exercised when interpreting the peripheral BDNF levels as direct indicators of brain activity. BDNF is produced in various tissues, including skeletal muscle, immune cells, and adipose tissue, all of which can contribute to the circulating levels, complicating the interpretation of blood BDNF as a marker of CNS function. Despite this, we sought to minimize the confounding factors by opting to measure BDNF in plasma rather than serum. The plasma BDNF levels provide a more reliable and sensitive indicator, as they contain a lower fraction of BDNF derived from platelets, which release BDNF upon activation [55]. This approach reduces the risk of BDNF level variability due to release from platelet stores. Another issue relates to other factors known to influence the blood BDNF levels, such as the presence of a circadian rhythm, with levels being significantly higher in the morning [38]. To mitigate this bias, we chose to collect blood samples from the patients and controls once, between 8:00 and 10:00 a.m. Lastly, it is important to consider how changes in DNA methylation in the blood can actually reflect changes in the target tissue (the brain). However, a strong correlation has recently been found between the methylation levels of the BDNF exon I promoter in the ventral prefrontal cortex and the quadriceps [56]. Moreover, similar epigenetic changes caused by environmental factors, particularly DNA methylation, in the brain and peripheral tissues in connection with psychiatric disorders have been reported, suggesting the possibility of investigating DNA methylation changes in the blood as putative biomarkers of psychiatric disorders [57,58].

In conclusion, the present study demonstrates higher pBDNF levels in patients suffering from AD and MDD than in healthy controls and a negative correlation in AD and MDD patients between job demands and BDNF promoters’ methylation. The above-mentioned limitations affect the relevance of our findings, which must be considered preliminary, needing validation in extended samples and in-depth analysis through longitudinal research. However, the results of the present study suggest that BDNF could play a key role in the pathophysiology of occupational stress-related disorders and that an elevation of its peripheral levels could contribute to protecting neural cells under stress conditions. The elevation of the pBDNF levels, even in MDD, may characterize a “reactive” subtype of major depressive episode, while the significant increase in the BDNF promoters’ methylation in depressed patients could express a predisposition to more severe illness under stress. Investigating the neuronal damage mediated by stress appears to be a real priority for preventive monitoring of workers exposed to occupational stress. Taking into account that work-related stress is reported to be an emerging risk involving a constantly increasing fraction of the working population, the results of the present study should solicit further research focusing on putative biomarkers for stress-related disorders as a potential tool for the diagnosis and prevention of occupational diseases.

## Figures and Tables

**Table 1:** Main features of the primers used in the methylation analyses are indicated (Ta: annealing temperature; amplicon size in base pairs (bp); number of CpG sites analyzed; gene sequence reference).

BDNF	Primer Sequence	Ta	Amplicon Length	CpG Number	Reference Sequence
BDNF-I	F 5′-GGGTTGTTAATTTATATTTGGGAAGT-3′ R 5′-ACCACTAATTACCCACAAAACC-3′	58 °C	119 bp	4	NG_011794.1 from 4557 to 4675
BDNF-IV	F 5′-GATTTTGGTAATTCGTGTATTAGAGTG-3′ R 5′-CATCAACTAAAAACTCCATTTAATCTC-3′	60 °C	212 bp	15	NG_011794.1 from 25326 to 25537

**Table 2:** Comparisons between patients and healthy controls (HCs) for the sociodemographic characteristics, stress rating scale scores, pBDNF and BDNF exon I/IV promoters’ methylation levels. Legend—AD: adjustment disorder, MDD: major depressive disorder, HC: healthy control, pBDNF: plasma brain-derived neurotrophic factor, JCQ: Job Content Questionnaire, BDI-II: Beck Depression Inventory-II, SAS: Self-rating Anxiety Scale. Significant *p*-values are shown in bold.

	AD Patients (n = 62)	MDD Patients (n = 79)	HCs (n = 44)	*p* Value
Age (mean ± SD)	47.63 ± 8.46	50.30 ± 8.00	47.10 ± 8.50	0.06
Gender M (%)	59.1	41.8	51.6	0.16
Education ≥ 13 years (%)	69.4	71.8	84.1	0.20
Public company workers (%)	38.7	51.9	26.8	**0.02** **(χ^2^ = 7.334)**
Private company workers (%)	61.3	48.1	73.2	**0.02** **(χ^2^ = 7.334)**
Temporary job (%)	1.6	3.8	22.0	**0.0001** **(χ^2^ = 17.747)**
Stable job (%)	98.4	96.2	78.0	**0.0001** **(χ^2^ = 17.747)**
pBDNF (pg/mL) (median [IQR])	69.1 [46.2–116.2]	55.7 [38.6–84.1]	32.5 [22.6–54.6]	**<0.000001**
BDNF exon I promoter’s methylation levels (%) (median [IQR])	3.3 [1.6–5.0]	6.7 [1.4–9.0]	3.0 [1.8–4.1]	**<0.001**
BDNF exon IV promoter’s methylation levels (%) (median [IQR])	0.2 [0.0–0.4]	1.06 [0.20–3.9]	0.2 [0.0–0.6]	**<0.000001**
JCQ decision latitude (median [IQR])	58.00 [50.00–70.00]	58.00 [49.50–64.50]	66.00 [60.50–73.50]	**<0.001**
JCQ job demands (median [IQR]))	35.50 [32.00–41.00]	43.00 [35.00–58.00]	32.00 [29.25–35.00]	**<0.000001**
JCQ social support (median [IQR])	16.50 [14.00–19.00]	26.00 [16.00–39.00]	24.00 [22.00–24.75]	**<0.000001**
BDI-II total score (median [IQR])	24.00 [18.75–43.00]	24.00 [20.00–29.5]	/	0.89
SAS total score (median [IQR])	49.00 [41.00–57.00]	50.00 [39.00–59.00]	/	0.89

**Table 3:** Correlation coefficients (r) between biological variables and JCQ scores in the AD and MDE patient groups. Legend—AD: adjustment disorder; MDD: major depressive disorder; HC: healthy control; pBDNF: plasma brain-derived neurotrophic factor; JCQ: Job Content Questionnaire. Significant *p*-values are shown in bold.

	AD Group (n = 62)			MDD Group (n = 79)			HC Group (n = 44)		
Variables	JCQ Decision Latitude Score	JCQ Job Demands Score	JCQ Social Support Score	JCQ Decision Latitude Score	JCQ Job Demands Score	JCQ Social Support Score	JCQ Decision Latitude Score	JCQ Job Demands Score	JCQ Social Support Score
**pBDNF levels**	0.134	−0.084	−0.129	−0.147	0.047	−0.061	0.043	0.094	0.247
**BDNF exon I promoter’s methylation levels**	0.122	−0.134	−0.112	−0.140	**−0.236** ***p* = 0.04**	**−0.244** ***p* = 0.03**	0.052	**0.374** ***p* = 0.01**	−0.056
**BDNF exon IV promoter’s methylation levels**	−0.030	**−0.295** ***p* = 0.02**	−0.053	0.049	**−0.588** ***p* < 0.0001**	**−0.686** ***p* < 0.0001**	−0.024	0.260	−0.166

## Data Availability

The data that support the findings of this study are available on reasonable request from the corresponding author. The data are not publicly available due to privacy and ethical restrictions.
